# Correction: Hydrothermal synthesis of nitrogen- and sulfur-co-doped carbon quantum dots from rice straw for fluorescent detection of Hg^2+^ in aqueous media

**DOI:** 10.1039/d6ra90038k

**Published:** 2026-04-07

**Authors:** Duc Cuong Nguyen, Bao Long Hoang, Van Duong Pham, Hop Bao Tung Nguyen, Tuan Anh Pham, Thi Thao Vu

**Affiliations:** a Faculty of Engineering Physics and Nanotechnology, VNU University of Engineering and Technology, Vietnam National University, Hanoi 144 Xuan Thuy Road, Cau Giay Hanoi 100000 Vietnam vtthao@vnu.edu.vn; b Institute of Physics, Vietnam Academy of Science and Technology 10 Dao Tan Street, Giang Vo Hanoi 100000 Vietnam

## Abstract

Correction for ‘Hydrothermal synthesis of nitrogen- and sulfur-co-doped carbon quantum dots from rice straw for fluorescent detection of Hg^2+^ in aqueous media’ by Duc Cuong Nguyen *et al.*, *RSC Adv.*, 2026, **16**, 10479–10494, https://doi.org/10.1039/D5RA09779G.

The authors regret that one of the affiliations (affiliation a) was incorrectly shown in the original manuscript. The corrected list of affiliations is as shown here.

The Royal Society of Chemistry apologises for these errors and any consequent inconvenience to authors and readers.

